# Sacral neuromodulation for constipation and fecal incontinence in children and adolescents – study protocol of a prospective, randomized trial on the application of invasive vs. non-invasive technique

**DOI:** 10.1186/s13063-024-08052-6

**Published:** 2024-03-22

**Authors:** Manuel Besendörfer, Annemarie Kirchgatter, Roman Carbon, Christel Weiss, Hanna Müller, Klaus E. Matzel, Sonja Diez

**Affiliations:** 1 Pediatric Surgery, Department of Surgery, Friedrich-Alexander-Universität (FAU) Erlangen-Nürnberg, University Hospital Erlangen, Loschgestraße 15, Erlangen, 91054 Germany; 2 Department of Medical Statistics, Biomathematics, and Information Processing, Medical Faculty, Mannheim of Heidelberg University, Theodor-Kutzer-Ufer 1-3, Haus 3, Ebene 4, Mannheim, 68167 Germany; 3 Neonatology and Pediatric Intensive Care, Hospital for Children and Adolescents, University of Marburg, Baldingerstaße, Marburg, 35043 Germany; 4 Department of Surgery, Section of Coloproctology, Friedrich-Alexander-Universität (FAU) Erlangen-Nürnberg, University Hospital Erlangen, Maximiliansplatz 2, 91054 Erlangen, Germany

**Keywords:** Sacral neuromodulation, Sacral nerve stimulation, Enteral neuromodulation, Functional constipation, Chronic refractory constipation, Hirschsprung’s disease, Fecal incontinence

## Abstract

**Background:**

A therapeutic effect of sacral neuromodulation (SNM) on fecal incontinence (FI) and quality of life has been proven in adults. SNM is, however, rarely used in pediatric cases. The aim of the study is to investigate effects of SNM in pediatric constipation in a prospective parallel-group trial.

**Methods:**

A monocentric, randomized, unblinded, parallel-group trial is conducted. SNM is conducted in the invasive variant and in an innovative, external approach with adhesive electrodes (enteral neuromodulation, ENM). We include patients with constipation according to the ROME IV criteria and refractory to conventional options. Patients with functional constipation and Hirschsprung’s disease are able to participate. Participants are allocated in a 1:1 ratio to either SNM or ENM group. Clinical data and quality of life is evaluated in regular check-ups. Neuromodulation is applied continuously for 3 months (end point of the study) with follow-up-points at 6 and 12 months. Findings are analyzed statistically considering a 5% significance level (*p* ≤ 0.05). Outcome variables are defined as change in (1) episodes of abdominal pain, (2) episodes of FI, (3) defecation frequency, (4) stool consistency. Improvement of proprioception, influence on urinary incontinence, quality of life and safety of treatment are assessed as secondary outcome variables. We expect a relevant improvement in both study groups.

**Discussion:**

This is the first trial, evaluating effects of neuromodulation for constipation in children and adolescents and comparing effects of the invasive and non-invasive application (SNM vs. ENM).

**Trial registration:**

The study is registered with clinicaltrials.gov, Identifier NCT04713085 (date of registration 01/14/2021).

**Supplementary Information:**

The online version contains supplementary material available at 10.1186/s13063-024-08052-6.

## Background

Sacral neuromodulation (SNM) has been proven to reduce fecal incontinence (FI), and to improve quality of life in adult patients [1]. Low-intensity electrical charges are placed in contact with the target nervous tissue to achieve pulsed depolarization of axons [2]. However, evidence-based knowledge on mechanisms of action and efficacy is still limited. It has been postulated that SNM primarily affects afferent nerve activity [2]. Beyond that, regions of learning in the central nervous system and even their neuroplasticity are sought to be affected [3].

Despite these limitations in the understanding of its mode of action, SNM is established as surgical treatment option in adult patients because of its clinical efficacy [4]. As a minimally invasive approach, it is applied in patients with refractory urological and proctological conditions. Small, rechargeable, and magnetic resonance imaging-safe devices have made the implantation suitable even for children and adolescents. However, SNM is currently only used in highly selected pediatric cases, which does not answer the need for further therapies in refractory cases. Promising results of singular case studies and very small population studies have not yet been transferred to prospective case–control trials to gain evidence-based results. Furthermore, a non-invasive SNM option was developed to offer a quick, cost-effective, and child-friendly approach. Positive effects of SNM on functional constipation in children who continue to be treated conservatively could have been observed [5], while they remain controversial in adults [6, 7]. In a review of 2011, Pauwels et al. observed no significant improvement of constipation in most of the included studies and proposes to apply SNM only in adult patients, refractory to treatment in order to potentially avoid more invasive treatment options [8]. Long-term outcomes of Gortazar de las Casas and colleagues revealed an efficacy of 38% in adult patients within a follow-up time of 5 years [9]. However, we see the justification of a potential efficacy of SNM on pediatric constipation due to the following point: We support the hypothesis of a unifying dysfunction of the enteral nervous system [10], which might even more connect functional/slow-transit-constipation (FC), rectal evacuation disorders, congenital neuro-intestinal dysfunctions with heterogeneous symptoms of constipation and fecal incontinence in children and adolescents. Indication for SNM in children and adolescents might therefore be discussed further.

There is currently a lack of randomized trials that are adequately powered and apply validated outcome measures, to allow for firm recommendations on the use of SNM in pediatric patients. We report a protocol of a monocentric, randomized, unblinded, parallel-group trial of SNM in pediatric and adolescent patients with symptoms of refractory chronic constipation.

## Materials and design

### Study objective and hypotheses

The aim of the study is to investigate the effects of SNM on symptoms and quality of life in children and adolescents. We intend to demonstrate that SNM is safe and effective in treatment of constipation and FI refractory to conventional therapeutic options.

The following hypotheses will be investigated within 3 months of treatment:

(A) SNM is superior to ENM regarding treatment of abdominal pain as main symptom of refractory chronic constipation in childhood and adolescence.
(B) Further exploratory hypotheses will be evaluated:
  SNM/ENM is effective and safe in reducing symptoms of chronic constipation and FI in children and adolescents as an additional therapeutic approach while continuing conventional treatment options unchanged.
  SNM/ENM treatment is effective in the subgroup of patients with Hirschsprung’s disease (HD).

### Technique

#### Enteral neuromodulation (ENM)

**Fig. 1:**
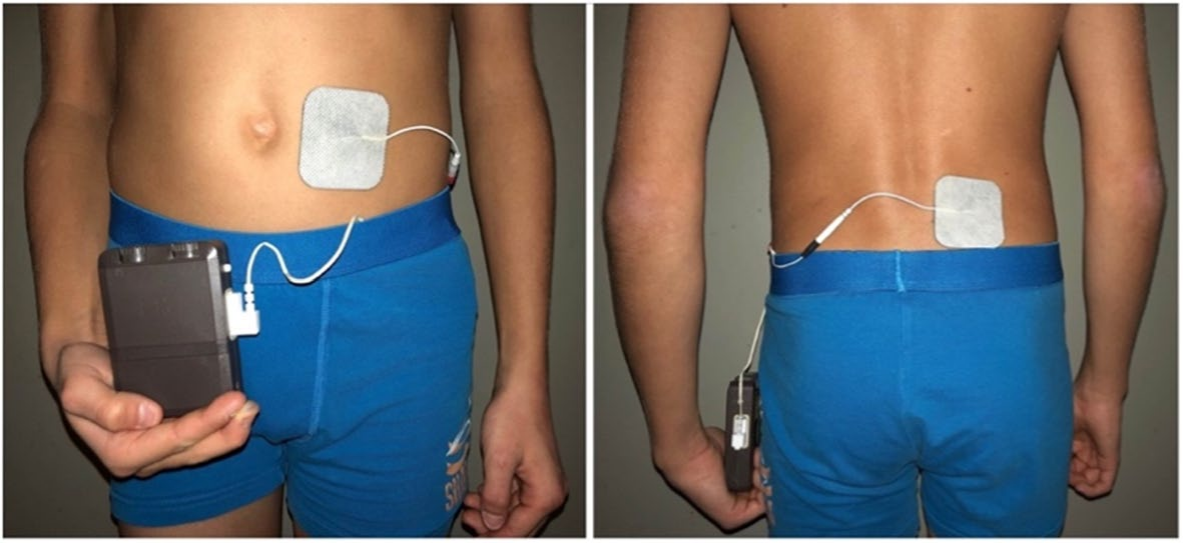
Enteral neuromodulation. Placement of the cutaneous adhesive electrodes of this non-invasive approach is depicted: the electrical field is built between a paravertebrally and periumbilically placed electrode

ENM is administered non-invasively via two cutaneous adhesive electrodes, placed paravertebrally and periumbilically (Fig. 1). The stimulation is applied with a frequency of 15 Hz and a pulse width of 210µs. Detailed stimulation techniques have been described previously [5] and are administered via a pulse generator (Ostimex® ProfiPlus TENS/EMS 335035) [11]. The adhesive electrodes (50 × 50mm in size) have a conductive surface and adhere to the skin by themselves. The manufacturer makes multiple use possible.

#### Sacral neuromodulation (SNM)

**Fig. 2:**
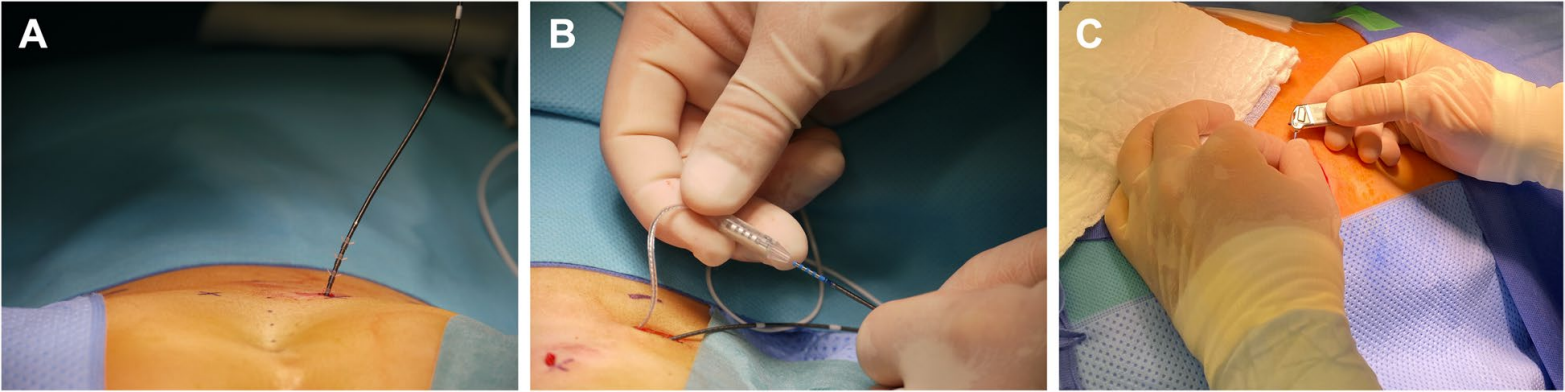
Sacral neuromodulation. Figure 2 illustrates the surgical implantation of sacral neuromodulation. **A** The tined lead electrode is placed at the sacral spinal nerve S3 or S4. **B**, **C** The previously implanted electrode is connected to a pulse generator, which is then implanted subcutaneously above the gluteal region

SNM is applied via two surgical interventions. First, the flexible electrode lead with four equally spaced electrode contact points (quadripolar tined lead electrode, Medtronic Interstim, Fig. 2A) is inserted percutaneously via the sacral foramen close to the sacral spinal nerve S3 or S4. A motor or sensory response can be monitored directly intraoperatively by probatory electrical stimulation and determines the side of the final implantation of the tined electrode. Stimulation is generated externally by a pulse generator and will be started on the first postoperative day with a frequency of 14 Hz and a pulse width of 210µs. The bipolar electrical field is generated between different poles of the tined lead, directly located at the sacral spinal nerves.

After a successful trial phase with a neuronal response, an implanted pulse generator (IPG, Medtronic® Interstim Micro) is connected to the implanted electrode (Fig. 2B) and subcutaneously implanted (Fig. 2C) 4 weeks after the electrode’s implantation to secure optimal electrode positioning and exclusion of postoperative complications.

### Trial design

**Fig. 3:**
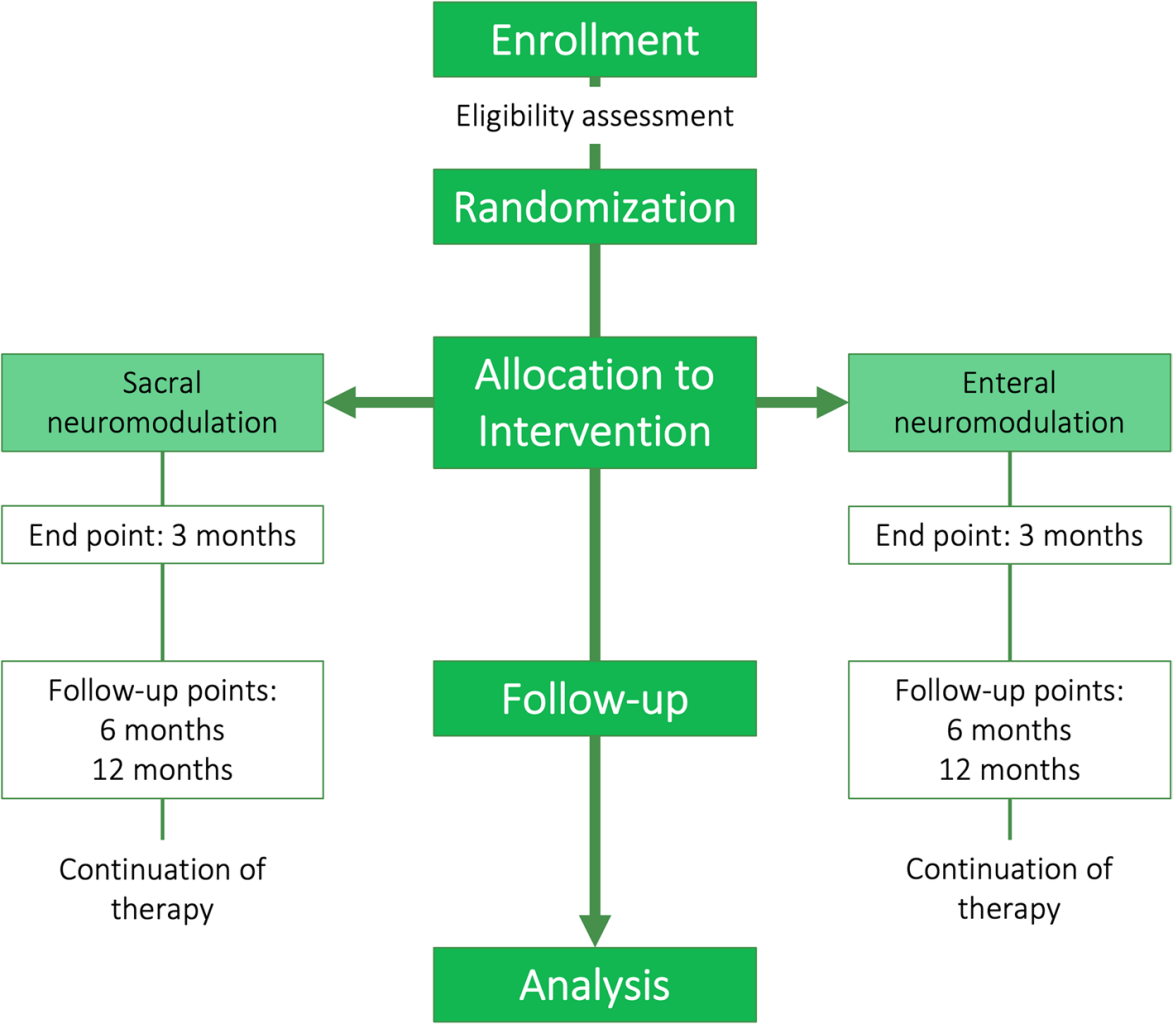
Study design. Figure 3 illustrates the design of the study, highlighting the comparison of the two study groups at different time points

This is a monocentric, randomized, unblinded, 2-arm parallel-group trial in a 1:1 allocation ratio. The study is conducted at the University Hospital Erlangen, Friedrich-Alexander-Universität Erlangen-Nürnberg (FAU) from 2019 to 2023. Potential subjects are screened among patients attending pediatric surgical and pediatric gastroenterological clinics. Diagnoses are made according to the ROME IV criteria for FC [12]. Interventions are provided to subjects based on their group assignment of either 3 months of SNM or ENM therapy. Regular checkups and a follow up appointment are conducted within the trial and after 6 and 12 months after the initiation of the intervention. All participants are informed of the purpose of the study and obtain their informed consent before participating in this parallel group study. The subjects may choose to withdraw from the study, or they may be withdrawn from the study, at any time at the discretion of the investigator. If a subject withdraws or is withdrawn, every effort is made to complete and report the observations. The study design is presented in Fig. 3.

### Study population: eligibility criteria

#### Inclusion criteria

To be eligible for participation, subjects are required to fulfill the following criteria:

- age between 2–17 years
- informed consent
- chronic constipation according to the ROME IV criteria for more than 3 months with abdominal pain and with or without FI [12] despite underlying diseases such as FC, rectal evacuation disorders or HD.
- refractory to conventional treatment in an appropriate weight-adapted application (training for bowel movements, lifestyle changes, pelvic floor training, pharmacological options)
- in cases of HD: diagnosis confirmed histologically by rectal biopsies and in case of resection of an aganglionic segment: period between surgery and SNM at least 1 year
- in cases of anorectal malformation or mechanical obstruction: post-surgical status: period between surgery and SNM at least 1 year

#### Exclusion criteria

Exclusion criteria are determined as follows:

- metabolic, inflammatory, and hormonal causes for chronic constipation
- toxic megacolon or further emergencies, which must be treated surgically
- sacral fractures or malformations prohibiting SNM access to target nerves
- inflammatory bowel diseases
- external rectal prolapse
- neuronal malignancies under medical and radiation therapy
- seizures

### Recruitment

**Fig. 4:**
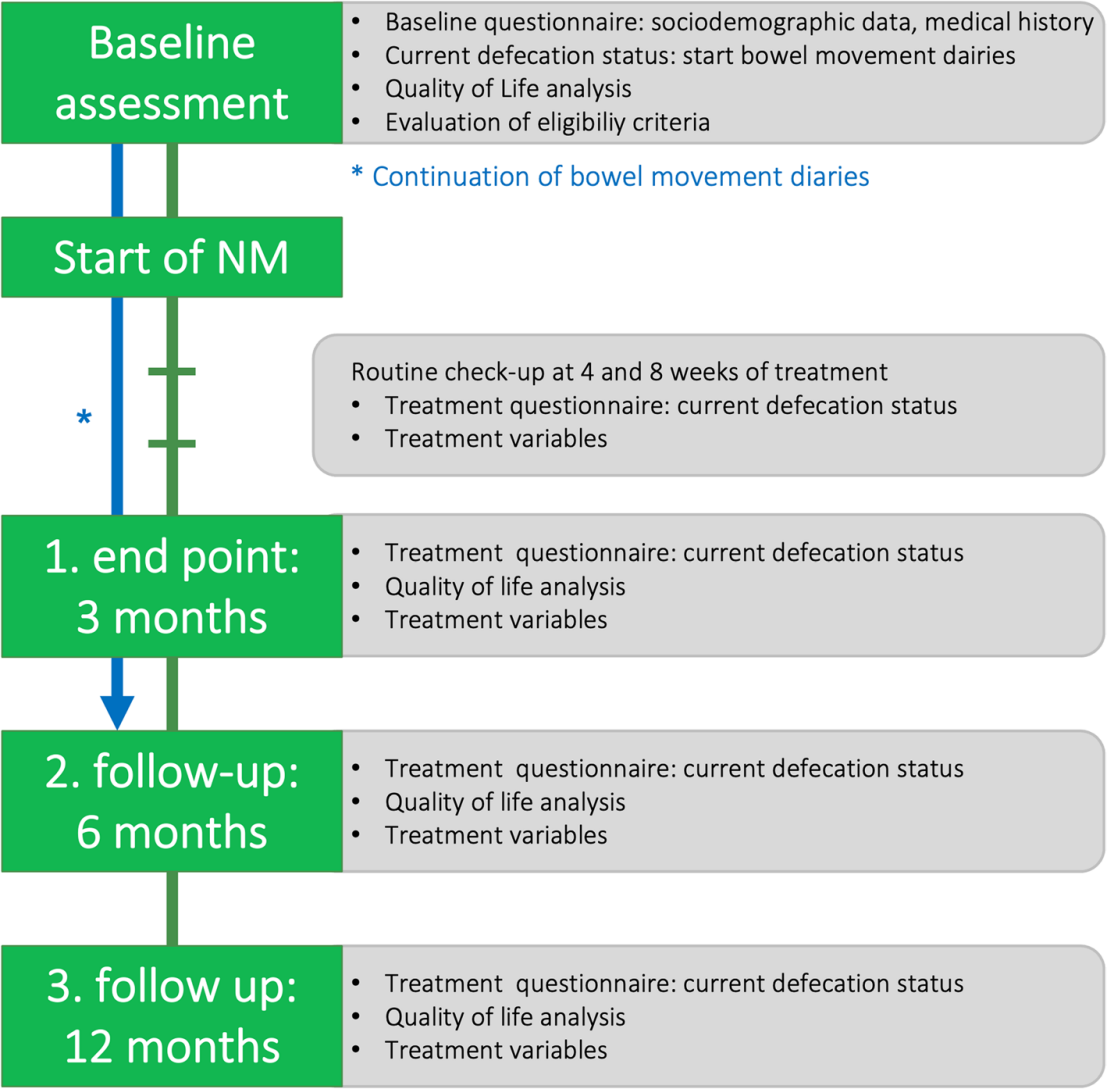
Study schedule. Figure 4 explains evaluation of outcome variables at different time points of the study

**Fig. 5:**
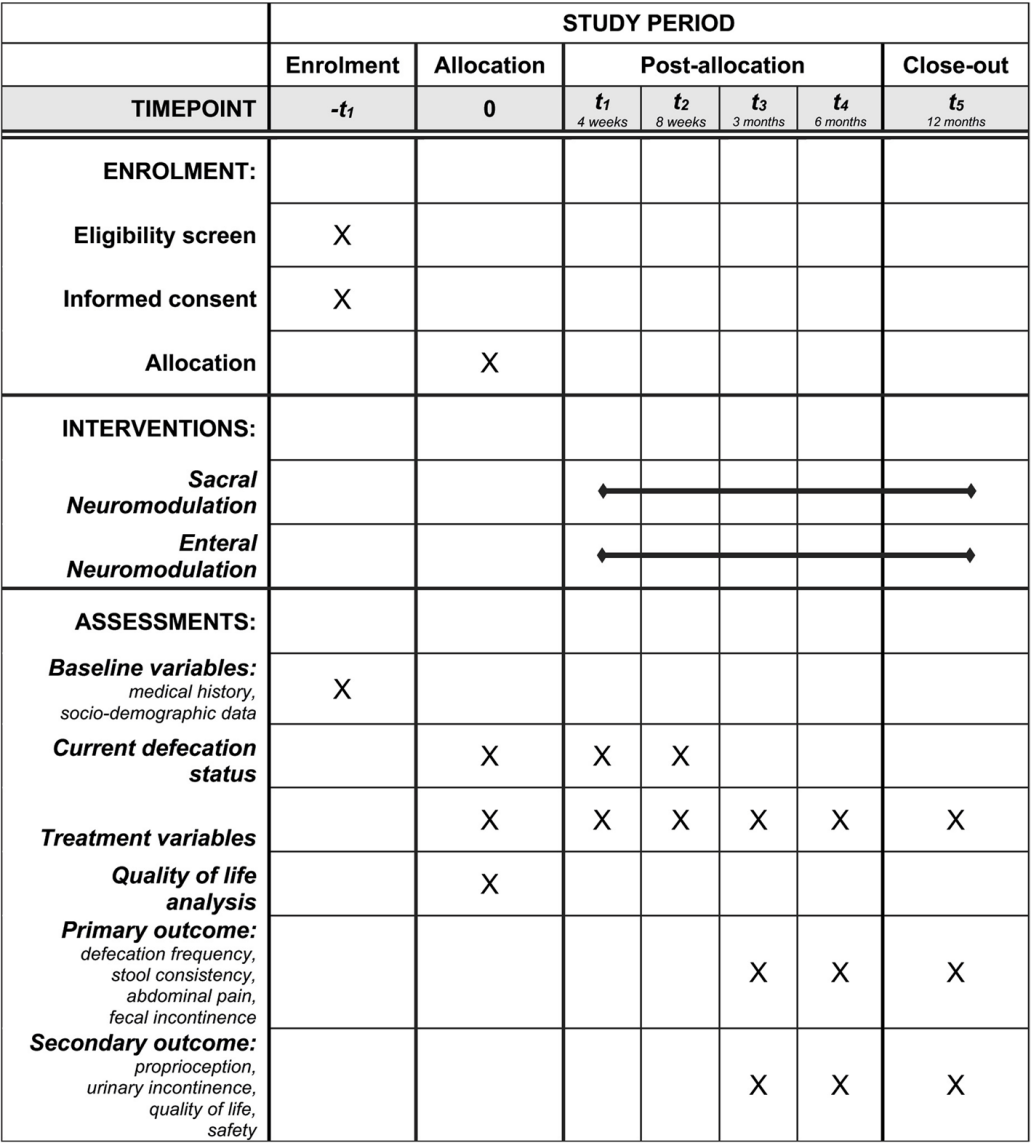
SPIRIT figure. Figure 5 summarizes the schedule of enrolment, interventions, and assessments according to the SPIRIT figure

Participants are recruited from the outpatients and inpatients clinics of the pediatric surgery and gastroenterology departments. Two or more experts determine the eligibility according to the inclusion and exclusion criteria. Informed consent, especially on treatment modalities and off-label use of SNM/ENM in childhood and adolescence, must be obtained before enrollment in the trial by next-of-kin and participants (6 years and older). Thereafter, a baseline assessment is performed, collecting all baseline information relevant to the study (sociodemographic data, medical history, current defecation status). The study schedule is summarized in Fig. 4 and included as the SPIRIT template as Fig. 5. At all timepoints, patients are able to withdraw their participation and end therapy with neuromodulation.

### Randomization

Participants are enrolled in the study groups at a 1:1 allocation to either the SNM or ENM group. Furthermore, patients are allocated 1:1 stratified based on HD/FC. Enrollment of subjects (assessment of eligibility, patient contact to obtain informed consent) is done by investigators and clinicians prior to randomization. The study design does not allow blinding of the therapist or further analyses in clinical follow-ups. The design is open label with only outcome assessors being blinded, so unblinding will not occur. Data analysis and statistical assessment are conducted separately and anonymously. The statistician performing the statistical analyses is blinded to individual group allocation and treatment.

### Interventions

#### Non-pharmacological treatment in both study groups

All subjects are provided with non-pharmacological management counseling prior to the study inclusion, including lifestyle modifications, such as (1) age-appropriate fluid consumption, (2) balanced diet and (3) regular sportive activity (at least 3 × 30min per week). Education and conduction of toilet training is further advised to improve regular bowel movements. If patients show further symptoms, irresponsive to these measurements, enrollment is conducted and non-pharmacological treatment is continued throughout the trial.

#### Pharmacological treatment in both study groups

Pharmacological options are included in the study population as follows: disimpaction with polyethylene glycol (PEG, 1.5-2mg/kg/d for 1–2 weeks) and initiation of maintenance therapy (PEG, 0.2–0.4 mg/kg/d) are conducted prior to the study inclusion. Maintenance therapy is continued throughout the study period in both groups. Supportive local applications such as saline enemas or stimulant laxatives (glycerin, bisacodyl) are applied as needed. Change of pharmacological treatment is not recommended during the study period, especially in case of rectal enemas. Medication due to other diagnoses is not changed during the study period.

#### Group 1: enteral neuromodulation

ENM is continuously applied for 12 weeks in each patient. Stimulation intensity is individually set by each patient to achieve a comfortable stimulation below pain threshold.

#### Group 2: sacral neuromodulation

With the implantation of the electrode, stimulation of S3/4 is continuously applied. Patients are able to set stimulation intensity parameters within a preset range via an external device with or without the IPG.

### Statistical methods

#### Objectivation and data validation

Specialized questionnaires were developed to objectivize symptoms and medical history of patients. These include sociodemographic data, medical history, and current defecation status and were adjusted for time points of “Baseline”, “Treatment with ENM” and “Treatment with SNM”.

Quality of life data are assessed according to the ‘Revised Children’s Quality of Life Questionnaire’ (KINDL^R^) before and after treatment. This reliable and validated questionnaire is a self-report measurement for health-related quality of life in children and adolescents [13, 14]. It consists of 24 5-point Likert-scale items, covering 6 quality of life dimensions: physical well-being, emotional well-being, self-esteem, family, friends, and daily functioning (school or nursery school/kindergarten). Items are partially reversely scored and linearly transformed to a 0 to 100 scale according to the manual. The sub-scales of these six dimensions are combined to produce a total score. Higher scores indicate a better quality of life. The questionnaire is available in three age-specific versions (The Kiddy-KINDL^R^ for 4–7 years of age, the Kid-KINDL^R^ for 8–12 years of age and the Kiddo-KINDL^R^ for 13–16 years of age).

#### Data collection

Data is extracted from the bowel movement diaries and specialized questionnaires. Recorded data is stored in case report forms at a secured place. Data is coded and entered in electronical files using Excel 2007 software (Microsoft Cooperation) by at least 2 different data administrators to reduce mistakes. All files are protected with password, which is only known by the investigators. Only the investigators have access to the final trial dataset. The information of the grouping and the results of the study are provided to the participants after the trial. Publications will only report aggregated data, and personal identities will not be disclosed.

#### Sample size calculation

As there is still insufficient clinical evidence on neuromodulation treatment in children and adolescents with FC and HD, this study requires only a small sample size. Power analysis is based on abdominal pain as primary outcome variable. A minimum of 78 subjects in total and 39 subjects per group is powered to detect a difference of at least 30%, with a 95% confidence interval (α = 0.05) and statistical power of 80% (β = 0.2).

#### Statistical analysis

IBM SPSS version 28 (IBM, Armonk, NY) is used to perform statistical analyses by an independent and blinded statistician. Continuous variables are presented as mean ± standard deviation, categorical data are reported as frequencies and percentages. If data losses occur during the study, the last observation is carried forward to adjust the missing data in follow-up evaluations. If large amounts of data are missing in one patient, the patient will be excluded from the study based on the decision of the project management group. If participant’s withdrawal is observed in a high number of patients, protocol modifications will be made in regular meetings. We compare clinical outcome data using chi-square and Fisher’s exact tests at defined time points pre- and post-treatment in both groups. Quality of life data is compared using unpaired or paired, two-tailed sample t-tests, if applicable. In case of multiple analyses, adjustment of p-values will be conducted accordingly. We set the confidence interval to 95% and all p-values less than 0.05 are considered indicative of statistical significance.

### Ethical approval and registration

The study protocol was approved by the local ethics committee (Friedrich-Alexander-Universität Erlangen-Nürnberg (FAU), No. B18_20) and complied with the Declaration of Helsinki. Written informed consent is obtained from each subject and next-of-kin by the investigators before the subject enters the trial. The study is registered with clinicaltrials.gov (Identifier NCT04713085). If protocol modifications are required, amendments will be submitted to and approved by the local ethics committee. To disseminate our findings, the clinical trial results will be published in peer-reviewed journals. Additionally, the study’s protocol is in congruence with the SPIRIT 2013 statement [15]; the SPIRIT checklist has been added to this publication.

### Organizational aspects of the trial

The coordinating project management group evaluates the trial’s progress and publishes necessary reports. Communication between ethics committee, patient groups and members of the trial’s staff is provided by the principal investigator, being the head of the coordinating project management group (meetings 4 per year). The trial steering committee coordinates organizational matters and documentational aspects (meetings 1 per month). Performing medical investigators meet once a month and are providing day by day support and patient’s contact. If protocol modifications are required, sponsors and funders are notified first by the project management group. The participating center will be informed based on a revised protocol, which will be sent as soon as the principal investigator, sponsors and funders are in agreement. Changed amendments will furthermore be submitted to and approved by the local ethics committee and updates will be uploaded at clinicaltrials.gov. Any deviations from the protocol will be fully documented using a breach report form. There are no external auditing trials planned, as this is a low-risk intervention.

In case of technical problems of applied devices with consecutive recalls, the trial is terminated by the principal investigator and interim results might then not be published.

### Patient and public involvement

The study supports patient and public involvement to improve the study’s design and outcome variables. Based on suggestions from patients and patients’ organizations at the beginning of the trial, inclusion criteria, documentation of data and outcome variables were modified. Further suggestions are repeatedly evaluated in regular meetings of the study’s head and possibly implemented as modification (see above).

### Study outcomes

Participants are required to complete bowel movement diaries throughout the trial to the first target point. Children aged up to 7 years are thereby represented by their next-of-kin, whereas participants ≥ 13 years of age are advised to fill in the questionnaires by themselves. Between age 8 to 12, documentation is conducted together and depending on the child’s autonomy. At routine check-ups at baseline, 4 weeks, 3 and 6 and 12 months, specialized questionnaires must be completed. Quality of life analysis is conducted at baseline and after 3, 6 and 12 months, as described below (Fig. 4).

#### Primary outcomes and measurement

Primary outcome variables are defined, and change is measured between time points of baseline, 3 and 6 months of therapy as follows:

1. Episodes of abdominal pain: Number of episodes of abdominal pain per week
  Definition of success: reduction by at least 50% of episodes per week
2. Episodes of FI: Number of episodes of FI per week
  Definition of success: reduction by at least 50% of episodes per week
3. Defecation frequency: Number of bowel movements per week
  Definition of success: doubling of episodes per week to at least 3 or more bowel movements per week
4. Stool consistency: Daily assessment of Bristol Stool Scale [16]
  Definition of success: change of at least 2 points within the scale of 1-7

Patients are classified based on treatment response and efficacy. A “clinically relevant” improvement is defined in cases with at least 2/4 fulfilled criteria, in which symptom control or -reduction is achieved.

#### Secondary outcomes and measurement

Secondary outcome variables are defined as follows:

1. Improvement of proprioception: as mentioned in the specialized questionnaires
2. Episodes of urinary incontinence: the number of episodes per week is evaluated with the criterion for a clinically relevant improvement in cases of reduction by at least 50% of episodes per week.
3. Quality of life: assessment based on the KINDL^R^ questionnaires
4. Safety of treatment: adverse events as mentioned in the specialized questionnaires

FI is diagnosed at the age of ≥ 4 years in cases of prior adequate toilet training and urinary incontinence at the age of ≥ 6 years with at least 4 episodes per week.

### Safety analysis and adverse events

Former studies on neuromodulation confirm a good safety profile. The participants conduct self-administered neuromodulation therapy at home. The participants are informed about potential adverse events and if any occur, they are instructed to terminate therapy and to immediately communicate with the researchers. Safety problems are reported to the clinical authorities (head of department, ethics committee) as well as to the manufacturers themselves. Appropriate treatment changes are then initiated. Adverse events are additionally recorded as part of the data collection for each session and will be reported to the clinical authorities and the manufacturers.

## Discussion

Neuromodulation is a promising approach in the treatment of chronic constipation and FI in children and adolescents. However, there is a substantial lack of well-planned, prospective clinical trials using an adequate sample size, appropriate methodology and validated outcomes to evaluate indications and efficacy in pediatric patients. Previously published outcomes of invasive and non-invasive sacral neuromodulation do predominantly not allow to draw firm conclusions because of small populations and high heterogeneity in outcome measures and participants. We were able to publish the first prospective randomized trial in 2022 on the efficacy of ENM (non-invasive SNM) in children and adolescents [11]. Patients, refractory to conventional treatment options, were included in the study and were randomized to either an optimization of these conventional options or to ENM under unchanged continuation of conventional options. We saw an improvement in outcome in 39% (conventional options alone) vs. 86% (ENM with conventional therapy, *p* < 0.0001). Conclusively, therapeutic efficacy of ENM could be shown, which serves as a baseline for this present study.

We here designed the study protocol of a prospective, monocentric, randomized 2-arm parallel-group trial, which to our knowledge is the first prospective, randomized study assessing SNM in pediatric patients and, above all, the first study assessing treatment differences between the invasive and non-invasive neuromodulatory approach (SNM vs. ENM).

We focused on determining appropriate sample size and reproducibility based on robust outcome variables. We also emphasized measurements to improve patient adherence, which is a challenge in pediatric clinical trials and especially regarding the use of self-administered therapy. The study design was therefore based on: (1) detailed information on mode of action, expected outcomes and self-administration for parents and in a child-centered approach for both, child and adolescent; (2) focus on the autonomous use of treatment by the child and the essential impact of parental monitoring; (3) individual documentation forms according to the age of the patient and separately for parents; (4) close monitoring to record treatment success and easy accessibility of the principal investigator/performing medical staff by e-mail for any problems that may arise.

We accept that the choice of our study design allows the following limitations:

1. Compared to other clinical trials on patients with Hirschsprung’s disease or functional constipation, this study is adequately powered with a comparably small population size.
2. We included patients despite underlying diagnoses of neural enteropathies, such as differentiation of slow-transit constipation, rectal evacuation disorders and aganglionic segments (Hirschsprung’s disease). We accept this as a potential bias of the study, as this heterogeneity might influence results and limit their value. Nevertheless, mechanisms of action of neuromodulation are not fully understood and seem to address multiple neuronal fibers [17]. Influence might be seen despite this heterogeneity based on neural dysfunctions, which unite all defecation disorders [10].
3. We chose a patient-centered focus and patient-reported outcomes, as we acknowledge the importance of patient’s assessment of treatment benefits. However, this leads to the acceptance of influence of continued conventional treatment options, and to the challenge of objectivation of diverse symptoms and individual perception, which may lead to substantial bias. Additionally, multiple analyses are restrictively valuable, as all p-values are only explorative and therefore not confirmatory.
4. We recognize that the bias due to a placebo effect can only be eliminated using a sham stimulation. To exclude any placebo effects, various considerations were taken when designing the study. In this first comparative prospective trial on pediatric neuromodulation, we accepted data of unblinded patients and treating physicians without sham stimulation, as it was recommended by the local ethics committee.
5. The study focuses on treatment effects of neuromodulation and does not address the long-term clinical effectiveness or cost-effectiveness of SNM and ENM.

We hope that our study will help raise awareness of the high percentage of pediatric patients with refractory chronic defecation disorders, who are not treated sufficiently with established conservative and surgical options and will offer these patients a valuable new treatment option. The outcomes of our study will therefore not only be of importance for affected patients, parents and treating physicians, but also for researchers, medical specialist societies and patients’ support groups, as it may complete the treatment algorithm of chronic defecation disorders in pediatric patients.

We aim at providing evidence-based conclusions as basis for the routine implementation of neuromodulation in children and adolescents with refractory chronic constipation. Funding has also been obtained to investigate outcome variables in long-term courses.

## Trial status

This is the presentation of study protocol version 1.4 (15 October 2020). Participants are recruited to start since July 2018. Data collection will be finished in December 2023 and study completion is expected to be December 2024. The study is funded by the Deutsche Gesellschaft für Koloproktologie e.V.. The principal investigator and all collaborating physicians have no potential conflict of interests.

### Supplementary Information

**Supplementary Material 1.** 

## Data Availability

The datasets used and/or analyzed during the current study are available from the corresponding author on reasonable request.

## Abbreviations

ENM: Enteral neuromodulation
FC: Functional constipation
FI: Fecal incontinence
HD: Hirschsprung’s disease
IPG: Implanted pulse generator
SNM: Sacral Neuromodulation
